# Wildlife Surveillance in North-Eastern Italy: Diagnostic Insights and Viral Detection in Gray Wolves and Golden Jackals

**DOI:** 10.3390/ani16213499

**Published:** 2026-11-05

**Authors:** Mery Campalto, Giammarco Assirelli, Andrea Simonetto, Elisa Mazzotta, Arianna Brandimarti, Marilena Carrino, Francesca Crescenti, Alda Natale, Letizia Ceglie, Laura Gagliazzo, Alessia Franzoso, Federica Obber, Nicola Pozzato

**Affiliations:** Istituto Zooprofilattico Sperimentale delle Venezie (IZSVe), Viale dell’Università 10, 35020 Legnaro, Italy; mcampalto@izsvenezie.it (M.C.); gassirelli@izsvenezie.it (G.A.); asimonetto@izsvenezie.it (A.S.); aribrandimarti@gmail.com (A.B.); mcarrino@izsvenezie.it (M.C.); fcrescenti@izsvenezie.it (F.C.); anatale@izsvenezie.it (A.N.); lceglie@izsvenezie.it (L.C.); lgagliazzo@izsvenezie.it (L.G.); afranzoso@izsvenezie.it (A.F.); fobber@izsvenezie.it (F.O.); npozzato@izsvenezie.it (N.P.)

**Keywords:** gray wolf, golden jackal, gross pathology, wildlife disease surveillance, infectious diseases, zoonosis, mammalian orthoreovirus, rotavirus, canine parvovirus (CPV2), feline parvovirus (FPV), canine coronavirus

## Abstract

Interfaces among humans, wildlife and domestic animals represent critical hotspots for the emergence and spread of infectious agents. The gray wolf (*Canis lupus*) and the golden jackal (*Canis aureus*) are expanding wild canid populations in Italy, whose increasing overlap with anthropized environments may facilitate pathogen transmission. This study investigated mammalian orthoreovirus (MRV), rotavirus A (RVA), canine coronavirus (CCoV), canine parvovirus (CPV2) and feline parvovirus (FPV) in wild canids from north-eastern Italy. The 101 carcasses (83 gray wolves and 18 golden jackals) obtained through passive wildlife surveillance were examined post-mortem. Tissue samples and swabs were screened using molecular assays, and positive samples were characterized and tested for virus isolation. MRV was detected in gray wolves (27.7%) and golden jackals (33.3%), representing the first report and successful isolation from both species. RVA prevalence was 60.2% in gray wolves and 61.1% in golden jackals. CCoV occurred exclusively in gray wolves (12.5%). CPV-2 was found in 10.8% of gray wolves and 35.3% of golden jackals, with FPV accounting for 66.7% of CPV-2-positive golden jackal samples. These findings provide valuable data on the circulation of MRV, RVA, CCoV, and CPV-2/FPV in free-ranging gray wolves and golden jackals and highlight the value of passive surveillance within a One Health framework overall.

## Simple Summary

Gray wolves and golden jackals are expanding their range across Italy, increasing contact with people and domestic animals. This overlap creates opportunities for viruses to circulate among wildlife, pets, livestock, and, in some circumstances, humans. Within a wildlife passive surveillance framework in north-eastern Italy, this study investigated four viral pathogens in 83 gray wolves and 18 golden jackals found dead and examined post-mortem. Tissue samples and swabs were screened using molecular methods. Mammalian orthoreovirus and rotavirus A were frequently detected in both species, whereas canine coronavirus, canine parvovirus 2 and feline parvovirus were detected less frequently. Notably, we isolated mammalian orthoreovirus from both gray wolves and golden jackals for the first time, providing material for future genetic investigations. In addition, we identified distinct canine parvovirus type 2 variants in gray wolves and detected feline panleukopenia virus in several golden jackals. Although carcass condition limited the pathological investigation, these findings provided new information on viruses circulating in free-ranging wild canids. These results enhance our understanding of disease ecology and highlight the importance of continuous wildlife surveillance within a One Health approach, recognizing the close links between animal, human, and environmental health.

## 1. Introduction

Interfaces between wildlife, domestic animals and humans are key points of pathogen emergence and spillover, especially within human-modified habitats [1,2]. In the Italian Alps, ecosystem recovery following decades of anthropogenic change has facilitated the recolonization of wild ungulates and carnivores, reshaping wildlife–human interactions and potentially creating new opportunities for pathogen transmission [3,4].

Wildlife species have progressively adapted to human presence, frequently exploiting roads, bridges and other infrastructure to access food and other resources, increasing contact opportunities with domestic animals and humans [5].

The gray wolf (*Canis lupus*) and the golden jackal (*Canis aureus*) represent useful models for investigating pathogen exchange, as their ongoing expansion into human-modified landscapes [6,7,8] increases contact with domestic animals and human activities [2,9].

Following a severe historical decline caused by habitat loss, prey reduction and persecution, the Italian wolf population reached a demographic minimum in 1973, when approximately 102 individuals were estimated to remain in small, fragmented groups across an area of about 8645 km^2^ in central and southern Italy [10].

In recent decades, however, gray wolves markedly recovered across Europe, supported by habitat regeneration associated with rural depopulation, increasing ungulate populations and reduced persecution under legal protection frameworks [4,6]. National monitoring in 2020–2021 estimated approximately 3307 gray wolves in Italy, including about 952 individuals in the Alpine region [11,12].

As the population expanded, gray wolves progressively moved from well-preserved habitats into more anthropized landscapes [13,14,15]. Public debate has largely centered on livestock and pet depredation [16,17,18] and on illegal retaliatory actions [19], yet from an epidemiological perspective, the increasing spatial overlap between gray wolves, domestic animals and human activities has created favorable conditions for pathogen circulation and cross-species transmission at the wildlife–domestic–human interface. Golden jackals, historically confined to Mediterranean and Black Sea coastal regions, have undergone a marked expansion in distribution and abundance across Europe during the 20th century [19,20,21]. Present in Italy since the 1980s, this species occupies a broad range of habitats, from lowland wetlands to mountain areas, and readily exploits suburban and peri-urban environments where anthropogenic food resources are available [7,8,22].

Competition with gray wolves may further influence their spatial distribution, potentially favor their presence closer to human settlements [23,24].

Domestic dogs may also act as bridging hosts because of their close taxonomic relationship with wild canids and their frequent presence in natural environments, potentially facilitating pathogen exchange between wildlife and human-associated settings [2,5,9].

Nevertheless, current knowledge of virus circulation in expanding wild canid populations remains limited, with few and often outdated studies available [25,26,27,28,29,30,31,32].

Given the wide range of pathogens potentially circulating at the wildlife–domestic animal interface, this study focused on mammalian orthoreovirus (MRV) and rotavirus A (RVA), both recognized as zoonotic agents but still poorly characterized in wild canid populations. Canine coronavirus (CCoV) and *Protoparvovirus carnivoran* 1, in particular canine parvovirus type 2 (CPV-2), two of the most prevalent viral pathogens of domestic dogs, were also included in the investigation.

MRV infections are typically associated with mild or asymptomatic enteric [33,34,35,36,37,38] and respiratory [39,40,41] disease in both humans and animals, although severe clinical manifestations, including encephalopathy [42] and meningitis [43,44], have also been reported. RVA represents a leading cause of acute gastroenteritis in humans and animals [45]. CCoV generally causes mild, self-limiting enteritis, although more severe disease may occur in coinfections, particularly with CPV-2 [46]. CPV-2 is a major cause of acute, potentially fatal gastroenteritis, particularly in puppies and immunologically susceptible dogs [47]. These clinical manifestations underscore the importance of monitoring viral circulation in wild canids, although infection does not necessarily result in overt disease. In a recent study [48], we provided molecular and virological evidence of MRV and RVA circulation in owned dogs and cats in Italy, highlighting their presence in animals living in close contact with humans. The objective of this study was to evaluate whether wild-canid cadavers collected through passive wildlife surveillance under official control and routinely supported by forensic pathology examination, could represent a suitable and informative source for assessing the circulation of selected viral pathogens within expanding wild carnivore populations.

## 2. Materials and Methods

### 2.1. Study Area and Study Population

The study was conducted in the Veneto Region and the Autonomous Provinces of Trento and Bolzano, within the territorial remit of the Istituto Zooprofilattico Sperimentale delle Venezie (IZSVe). Gray wolves and golden jackals submitted through passive wildlife surveillance programs (European Animal Health Law (Regulation 2016/429) and Directive 2003/99/EC on zoonoses) between May 2024 and January 2026 were included through opportunistic, event-driven sampling. Carcasses in an advanced state of decomposition or lacking the required matrices were excluded. All animals tested negative for rabies virus prior to necropsy.

### 2.2. Diagnostic Approach and Sample Collection

When carcass conditions allowed, sex and age class were recorded for each animal. Age class was determined based on body structure, weight and tooth eruption patterns, with individuals classified as juveniles (<1 year), sub-adults (1–2 years) and adults (>2 years) [49].

All carcasses were examined according to a standardized protocol in line with Italian national guidelines for forensic veterinary necropsy [50] to determine the cause of death. Necropsies of both species were performed by the same team, ensuring consistency in examination procedures and sample collection. Furthermore, all samples were georeferenced according to their place of recovery or geographic coordinates recorded using a WebGIS platform IZSVe; maps were generated with QGIS software (software version Quantum Gis 3.44).

Each animal first underwent an external physical examination to assess nutritional status, muscle-skeletal development, mucous membranes, and any alterations to the skin or skin appendages. Subsequently, carcasses were skinned, body cavities were opened and organs were extracted and examined individually. For each animal, during the necropsy, oropharyngeal and rectal swabs were collected, as well as tissue samples from the lungs, spleen, ileum, and mesenteric lymph nodes. Gross pathological findings were documented, with a particular focus on the gastrointestinal tract and lymphoid organs.

Samples were stored at +4 °C and promptly delivered to the IZSVe Diagnostic Virology Laboratory for molecular analyses.

All collected matrices were analyzed for MRV and RVA. In addition, as part of the routine diagnostic workflow, lung and ileum samples were tested for CCoV, while ileum samples were analyzed for CPV-2.

### 2.3. Molecular Investigation

#### 2.3.1. Sample Preparation and Nucleic Acids Extraction

A portion of tissue samples (~5 mm^3^) was homogenized in 0.8 mL of 1X Phosphate-Buffered Saline (PBS) supplemented with antibiotics (10,000 IU/mL penicillin G, 10 mg/mL streptomycin, 5000 IU/mL nystatin, and 0.25 mg/mL gentamicin sulfate) (PBS-A) using steel beads in a TissueLyser (QIAGEN, Hilden, Germany) at 30 Hz for 3 min (min). Swabs were cut in sterile microtubes containing 1 mL of PBS-A, thoroughly vortexed, and briefly centrifuged. One hundred microliters (µL) of each supernatant were subjected to viral nucleic acid extraction using the ID Gene^®^ Mag Universal Extraction Kit (IDvet, Grabels, France) on the KingFisher™ Flex Purification System (Life Technologies, Carlsbad, CA, USA), according to the manufacturers’ instructions. To monitor extraction efficiency and validate negative results, a universal heterologous RNA internal control (Intype IC-RNA, Generon S.p.A., San Prospero, Italy) was added to each sample at a ratio of 1:10 relative to the total elution volume and co-amplified [51]. Eluted nucleic acids were stored at −80 °C until use.

#### 2.3.2. Molecular Assays

Eluted RNA was screened using a multiplex real-time reverse transcriptase PCR (RT-qPCR) assay targeting conserved regions of the MRV L1 gene and the RVA NSP3 gene, with reaction setup and thermal cycling conditions applied as previously described by Campalto et al. (2026) [48], allowing the simultaneous detection of both viruses together with co-amplification of the Intype IC-RNA internal control.

As part of the routine diagnostic workflow, samples were also screened for CCoV and CPV-2. RNA was tested for CCoV using a ready-to-use RT-qPCR assay (VetMAX™ FIP Dual IPC Kit; Thermo Fisher Scientific, Waltham, MA, USA). MRV/RVA amplification was performed on a QuantStudio™ 5 Real-timePCR System (Thermo Fisher Scientific, Waltham, MA, USA), whereas CCoV amplification was carried out on a CFX96 Deep Well Real-time PCR System (Bio-Rad Laboratories Inc., Hercules, CA, USA).

In parallel, extracted DNA was analyzed by real-time PCR (qPCR) targeting a conserved region of the VP2 gene shared by CPV-2 and FPV, using an assay developed at the IZSVe, with reaction conditions and thermal profile as previously reported by Carrino et al. (2022) [52]. Each PCR run included a positive control, a negative extraction control to monitor potential contamination during nucleic acid extraction, and a no-template control (NTC).

In addition, samples positive for CPV-2 were further characterized by variant typing using a duplex fluorescence melting curve analysis (FMCA), based on the method described by Liu et al. (2019) [53], with minor modifications. Primer and probe concentrations were maintained as originally described, while the PCR master mix, thermal cycling conditions, and instrumentation were adapted to the experimental setup used in this study. Reactions were performed in a final volume of 25 µL, containing 12.5 µL of iTaq Universal Probes Supermix (Bio-Rad Laboratories Inc., Hercules, CA, USA), 0.25 µL of primer 87f-1 (1 µM), 0.25 µL of primer 87r-jb (10 µM), 0.75 µL of primer cpv-F2 (1 µM), 0.75 µL of primer ab-Rev (10 µM), 0.25 µL of probe P1 (10 µM), 0.75 µL of probe P2 (10 µM), 5 µL of template DNA, and nuclease-free water to volume. Amplification and melting curve analysis were carried out on a CFX96 Deep Well Real-Time PCR System (Bio-Rad Laboratories Inc., Hercules, CA, USA). The thermal profile consisted of an initial denaturation at 95 °C for 60 s, followed by 55 cycles of 95 °C for 15 s and 50 °C for 30 s (data acquisition). This was followed by a denaturation step at 95 °C for 180 s and a hybridization step at 37 °C for 180 s. Melting curve analysis was performed from 37 °C to 97 °C with a ramp rate of 0.5 °C/s and continuous fluorescence acquisition. Each run included reference controls for CPV-2a, CPV-2b, CPV-2c, and the original CPV-2 strain, along with a NTC. Melting profiles were interpreted according to the criteria described by Liu et al. (2019) [53].

Primers and probes used in the molecular assays are reported in Supplementary Table S1.

Samples identified as CPV-2 original by FMCA were further selected for Sanger sequencing of a portion of the VP2 gene, allowing discrimination between canine parvovirus type 2 and feline panleukopenia virus (FPV) strains.

Conventional end-point PCR was performed using the primer pair F2 (5′-GGATTTCTACGGGTACTTTC-3′, sense) and R2 (5′-AAACTTTAGTTGGTGGCTGAG-3′, antisense), as previously described [54]. PCR reactions were carried out in a final volume of 25 µL containing 5 µL of PrimeSTAR GXL Buffer (5X) (Takara, Kusatsu, Japan), 1.25 µL of each primer (10 µM), 2 µL of dNTP mix (2.5 mM), 0.5 µL of PrimeSTAR GXL DNA Polymerase (1.25 U/µL) (Takara, Kusatsu, Japan), 10 µL of sterile ultrapure water, and 5 µL of template DNA.

Amplification was performed on a ProFlex PCR System (Thermo Fisher Scientific, Carlsbad, CA, USA) under the following cycling conditions: an initial denaturation at 94 °C for 5 min, followed by 35 cycles of 94 °C for 30 s, 50 °C for 30 s, and 72 °C for 30 s, with a final extension at 72 °C for 8 min.

PCR products were analyzed by capillary electrophoresis using the LabChip^®^ GX Touch™ system (Revvity Inc., Waltham, MA, USA). Positive amplicons were subsequently subjected to Sanger sequencing using the same primers employed for amplification. Raw sequence data were processed using SeqScape v3, and the obtained sequences were compared with reference sequences available in GenBank using MEGA 12.0 [55].

### 2.4. Mammalian Orthoreovirus Isolation

Virus isolation was attempted primarily from all MRV RT-qPCR-positive samples, as its detection in gray wolves and golden jackals represented a novel finding and the recovery of infectious virus was considered valuable for further characterization.

The same specimens processed for molecular analyses were used, following the protocol previously described [48]. Tissue homogenates were diluted 1:5 in PBS-A, whereas rectal swab suspensions and ileum samples were diluted 1:10. After overnight incubation at 4 °C, clarified suspensions were passed through a 0.45 μm membrane filter and inoculated onto African green monkey kidney (Vero) cells (ATCC CCL-81). Vero cells were cultured in Minimum Essential Medium (MEM) supplemented with 10% fetal bovine serum, 2 mM L-glutamine, 1% penicillin–streptomycin, 0.5% gentamicin, 1% nystatin, and 0.1% amphotericin B. Subconfluent monolayers grown in 12.5 cm^2^ flasks were washed twice with PBS and inoculated with 400 µL of filtered sample mixed with 100 µL of MEM containing 5 µg/mL TPCK-treated trypsin. Following adsorption for 1.5 h at 37 °C in a 5% CO_2_ atmosphere, 4.5 mL of maintenance medium supplemented with 1 µg/mL TPCK-treated trypsin was added. Cultures were then incubated at 37 °C in 5% CO_2_ and monitored daily for cytopathic effect (CPE).

### 2.5. Statistics

A descriptive analysis was performed to explore data distribution, using the individual wild canid as statistical unit. Virological test results were assessed for MRV, RVA, CCoV, and CPV-2.

For each pathogen, prevalence was estimated as the proportion of positive animals over the total number of tested animals, with 95% confidence intervals calculated using the Wilson score method. Prevalence estimates were stratified by species, sex, and age class. A summary prevalence table was additionally generated, presenting results stratified simultaneously by species and age-class, as well as by species and sex.

To assess potential associations between the results of each molecular analysis and the corresponding gross pathology descriptors related to intestinal content and lymphoid organ reactivity, Fisher’s exact tests were performed. Fisher’s exact tests were also used to compare the prevalence of each pathogen across species, sex and age class. Raw *p*-values were adjusted for multiple comparisons using the Holm–Bonferroni method. All statistical analyses were performed using R (version 4.5.2).

## 3. Results

### 3.1. Study Population

During the study period, a total of 101 animals were examined, including 83 gray wolves and 18 golden jackals.

Among the gray wolves, most individuals originated from the Veneto region (*n* = 46, 55.4%), followed by the Autonomous Province of Trento (*n* = 30, 36.1%) and the Autonomous Province of Bolzano (*n* = 7, 8.4%). The population included 53 males (63.9%) and 30 females (36.1%) and was mainly composed of sub-adults (*n* = 44, 53.0%), followed by adults (*n* = 25, 30.1%) and juveniles (*n* = 14, 16.9%). The golden jackal individuals were equally sourced from the Autonomous Province of Bolzano (*n* = 9) and the Veneto Region (*n* = 9). The cohort included 10 males (55.6%) and eight females (44.4%), consisting primarily of sub-adults (*n* = 9, 50.0%), followed by adults (*n* = 6, 33.3%) and juveniles (*n* = 3, 16.7%).

**Figure 1:**
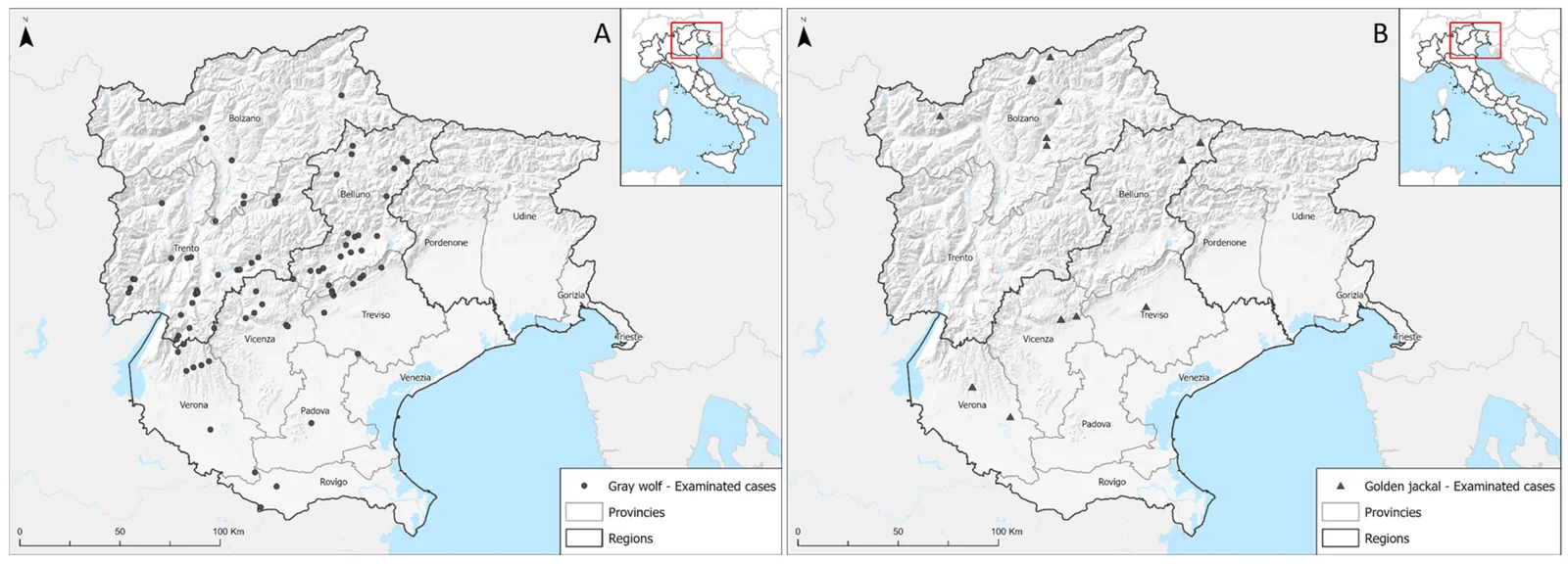
Geographical distribution of the sampled gray wolves (*Canis lupus*) (**A**) and golden jackals (*Canis aureus*) (**B**) recovered in north-eastern Italy. Animals were georeferenced according to their place of recovery or, when available, to the geographical coordinates recorded using the IZSVe WebGIS platform. Maps were generated using QGIS software.

The geographical distribution of the sampled gray wolves and golden jackals is shown in Figure 1A and Figure 1B, respectively.

### 3.2. Gross Pathology Findings

Gross gastrointestinal system findings, particularly intestinal content consistency, lymphoid tissue alterations and the presence of intestinal cestodes are summarized in Table 1.

### 3.3. Viruses Molecular Investigation

**Figure 2:**
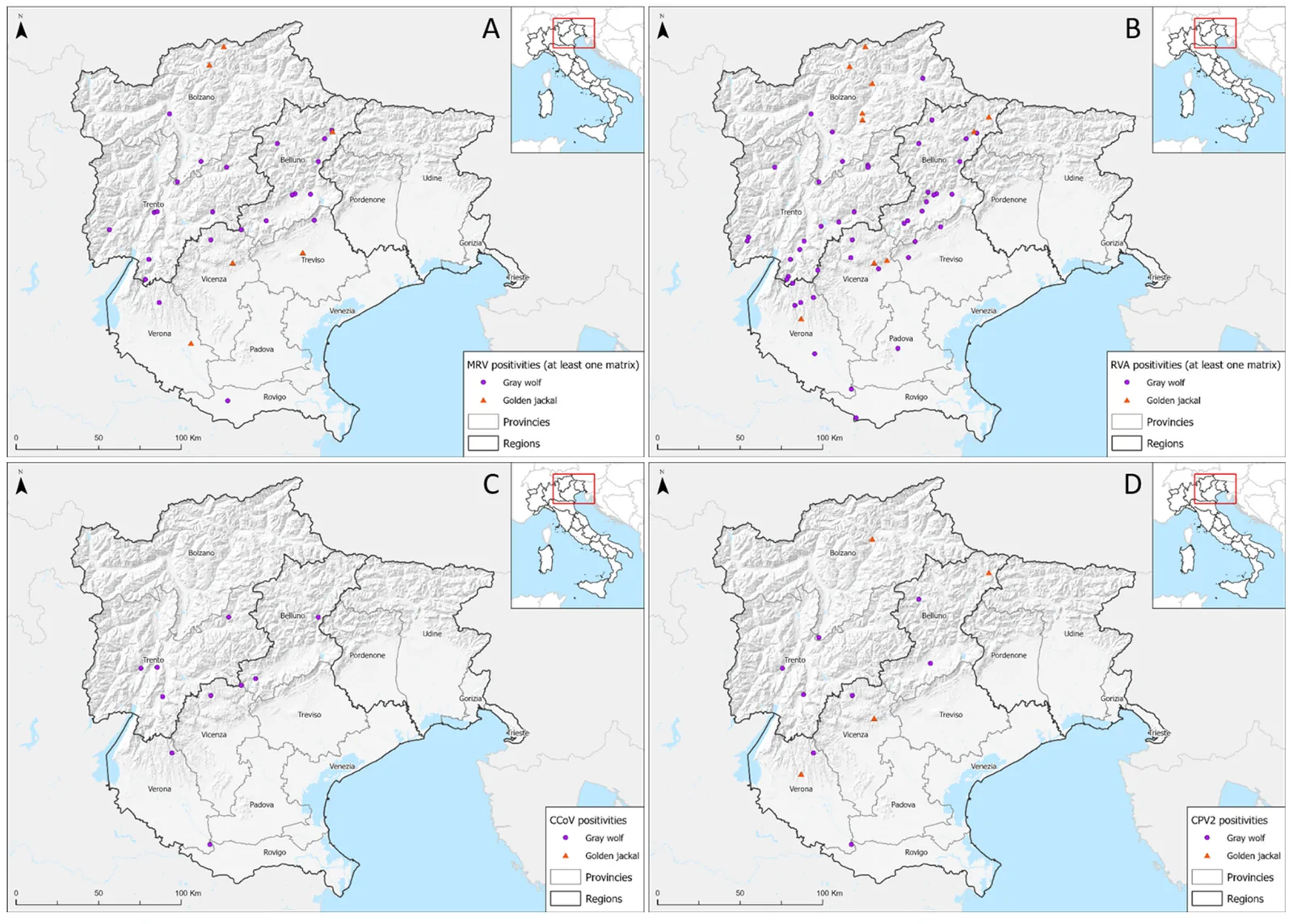
Spatial distribution of positive wild canids in north-eastern Italy. Panels show the geographic distribution of animals testing positive for (**A**) mammalian orthoreovirus (MRV), (**B**) rotavirus A (RVA), (**C**) canine coronavirus (CCoV), and (**D**) canine parvovirus type 2 (CPV-2), based on detection in at least one analyzed matrix. Each point represents a positive individual. Purple dots indicate gray wolves (*Canis lupus*), while orange dots indicate golden jackals (*Canis aureus*). The inset map highlights the study area within Italy.

The spatial distribution of positive cases is shown in Figure 2A–D.

MRV was detected in 23/83 (27.7%, 95%CI: 19.2–38.2) gray wolves and in 6/18 (33.3%, 95%CI: 16.3–56.2) golden jackals (Figure 2A). In gray wolves, positive matrices included ileum (17/83), mesenteric lymph nodes (14/83) and rectal swabs (3/83). Concurrent positivity in ileum and mesenteric lymph node was observed in nine animals, two of which were also positive in rectal swabs. In golden jackals, MRV was detected exclusively in ileum and mesenteric lymph nodes (5/18 each), with concurrent positivity of these matrices in four animals. All other investigated matrices tested negative.

RVA was detected in 50/83 (60.2%, 95%CI: 49.5–70.1) gray wolves and in 11/18 (61.1%, 95%CI: 38.6–79.7) golden jackals (Figure 2B). In gray wolves, positive matrices included mesenteric lymph nodes (40/83) and ileum (36/83), with concurrent positivity in 26 animals, whereas all rectal swabs were negative. In golden jackals, RVA was detected in ileum (11/18), mesenteric lymph nodes (9/18), lung (1/18), and oropharyngeal swab (1/18). Concurrent positivity was observed in nine animals, one of which was also positive in lung and oropharyngeal swab.

All MRV and RVA samples yielded valid results on molecular assays.

CCoV RNA was detected in 10/80 evaluable gray wolf ileum samples (12.5%, 95% CI: 6.9–21.5), while three additional samples were unsuitable for analysis due to advanced post-mortem changes (Figure 2C). All lung samples from gray wolves and all ileum and lung samples from golden jackals tested negative.

CPV-2 was detected in 8/74 gray wolf (10.8%, 95% CI: 5.6–19.9) and in 6/17 golden jackal evaluable ileum samples (35.3%, 95% CI: 17.3–58.7) (Figure 2D). Overall, 66 gray wolves and 11 golden jackals tested negative, whereas 9 gray wolves and 1 golden jackal ileum samples were unsuitable for analysis due to poor carcass preservation.

CPV-2 variant typing was successfully performed on 14 positive samples, including eight from gray wolves and six from golden jackals. Among gray wolf samples, one was identified as CPV-2a, four as CPV-2b, one as CPV-2c, and one as original CPV-2. One additional gray wolf sample could not be typed by either FMCA or conventional PCR and Sanger sequencing. All six golden jackal samples were classified as original CPV-2 by FMCA [53]. Overall, seven samples identified as original CPV-2 (one gray wolf and six golden jackals) were subjected to conventional PCR targeting a portion of the VP2 gene [54]. Amplicons suitable for Sanger sequencing were obtained from four of the seven samples. Sequence analysis revealed that all four successfully sequenced samples, originating from golden jackals, were FPV.

Statistical analysis did not reveal any significant difference in the distribution of any infectious agent based on species, age-class, sex, lymph nodes reactivity and intestinal content. For the doubly stratified analysis (Table 2 and Table 3), formal statistical testing was not feasible due to the very small number of observations in several subgroups, resulting in contingency tables with expected cell counts too low to yield reliable results. Regarding the distribution by age-class, no consistent difference was observed across adult, sub-adult, and juvenile individuals in either species (Table 2).

Regarding the distribution of positivity by sex, prevalence estimates were broadly comparable between males and females within both species for all four pathogens, with RVA recording the highest positivity rates across all groups (gray wolf: 66.7% in females, 56.6% in males; golden jackal: 75.0% in females, 50.0% in males), while CCoV was completely absent in golden jackals of both sexes (Table 3).

### 3.4. MRV Isolation Findings

Virus isolation was attempted for all samples testing positive for MRV by multiplex RT-qPCR (23/83 gray wolves and 6/18 golden jackals). MRV was successfully isolated from two wild canids, i.e., one gray wolf and one golden jackal. To the best of our knowledge, this represents the first reported isolation of MRV from these wild canid species. In the gray wolf, MRV was recovered from multiple matrices, including ileum, mesenteric lymph node, and rectal swab, whereas in the golden jackal, virus isolation was successful from the mesenteric lymph node.

Cytopathic effects (CPE) in the gray wolf-derived isolate became evident after the third passage, while in the golden jackal-derived isolate clear CPE was already observed at the second passage. When no visible CPE was detected, up to three blind passages were performed.

MRV isolates were confirmed by molecular assays and whole-genome sequencing using next-generation sequencing (NGS). Detailed genomic characterization and phylogenetic analyses are beyond the scope of this study and will be reported in a separate study.

### 3.5. Case Reports: Diagnostic Description of a Gray Wolf and a Golden Jackal

An integrated evaluation of gross pathology and laboratory diagnostics allowed the identification of significant lesions directly attributable to the target viral pathogens in two specific cases (one gray wolf and one golden jackal).

#### 3.5.1. Case 1

A sub-adult male gray wolf (designated as “Subject 1”) from the Verona province was found at the border between a wooded and agricultural area, in close proximity to a road and residential buildings. On presentation, the animal was in good nutritional condition and showed moderate post-mortem changes. External examination revealed mild tick infestation, diffuse congestion of the accessible mucous membranes, and marked perineal fecal staining. No major traumatic injuries were observed on external examination or during skinning, apart from two focal hemorrhagic suffusions on the dorsum and rump. No lesions of the musculoskeletal system were detected.

**Figure 3:**
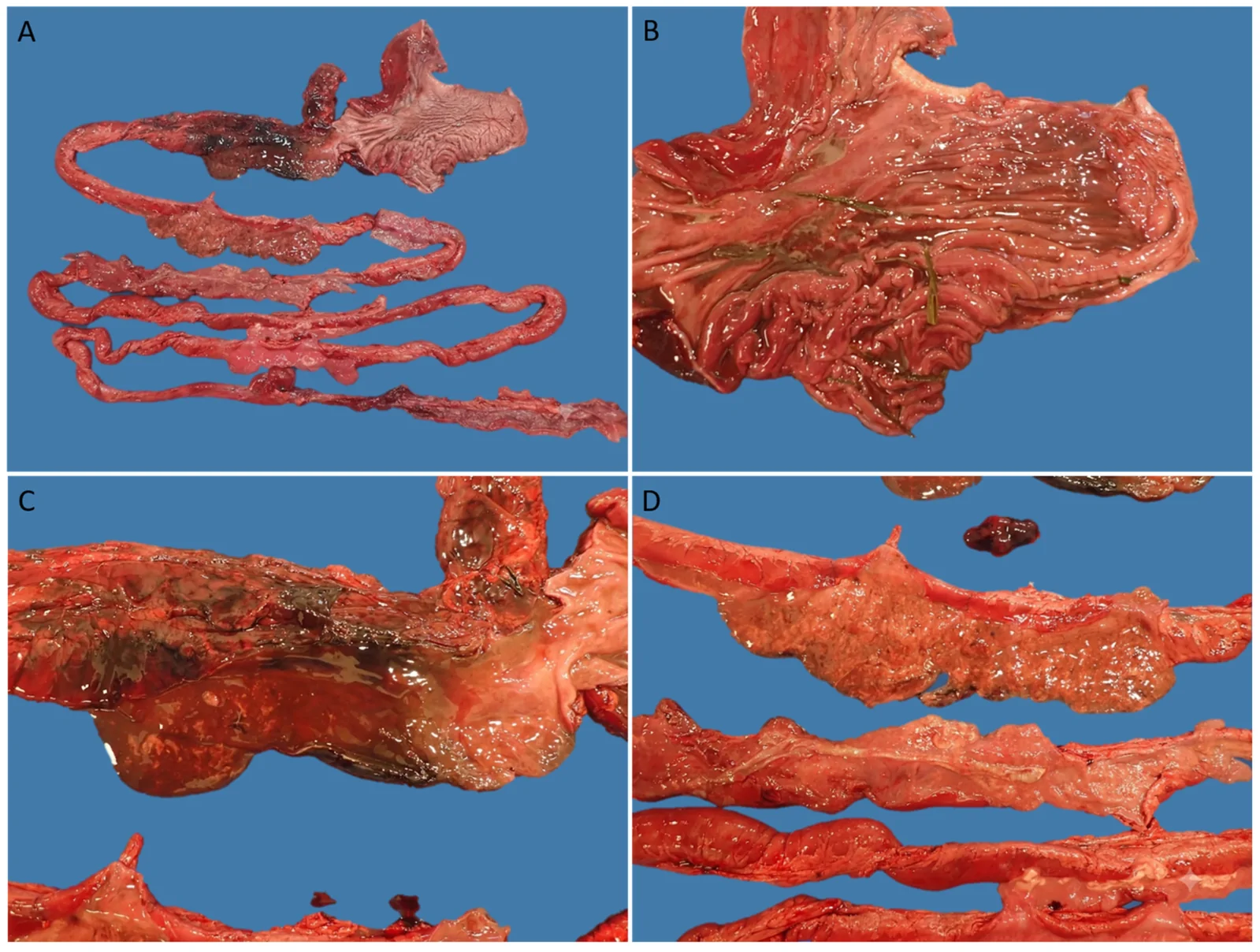
Macroscopic gastrointestinal findings in the gray wolf (Subject 1). (**A**) View of the entire gastrointestinal tract, which contains abundant red-to-brick-red, fluid-to-mucoid material. (**B**) Close-up of the stomach revealing diffuse mucosal hyperemia and edema. (**C**) Close-up of the duodenum showing severe, diffuse mucosal hyperemia and the same fluid-to-mucoid, red content. (**D**) Close-up of the jejunal lumen containing mucoid, dark-pink-to-brown material and a concurrent cestoda infestation.

In the respiratory and cardiovascular systems, there was severe diffuse pulmonary edema and congestion, with abundant foam filling the lower airways, together with mild bilateral hyperemia and edema of the atrioventricular valves and of the aortic semilunar valves. Severe lesions were present throughout the gastrointestinal tract (Figure 3A). The stomach contained abundant red fluid, and the gastric mucosa showed marked diffuse hyperemia (Figure 3B). The small intestine was filled with abundant fluid to mucous, red-to-brick-red content, with diffuse mucosal hyperemia (Figure 3C), a moderate cestode infestation in the jejunum (Figure 3D), and prominent Peyer’s patches in the ileum. In the large intestine, the content was scant, unformed, and red to purple, with minimal mucosal changes. These enteric changes were accompanied by severe congestion and enlargement of the mesenteric lymph nodes. The thymus and spleen were also markedly congested and enlarged. The kidneys showed diffuse parenchymal congestion, particularly evident at the corticomedullary junction, and the urinary bladder was empty.

Molecular analyses identified a viral co-infection of the ileum involving RVA, CCoV, and CPV-2b.

#### 3.5.2. Case 2

**Figure 4:**
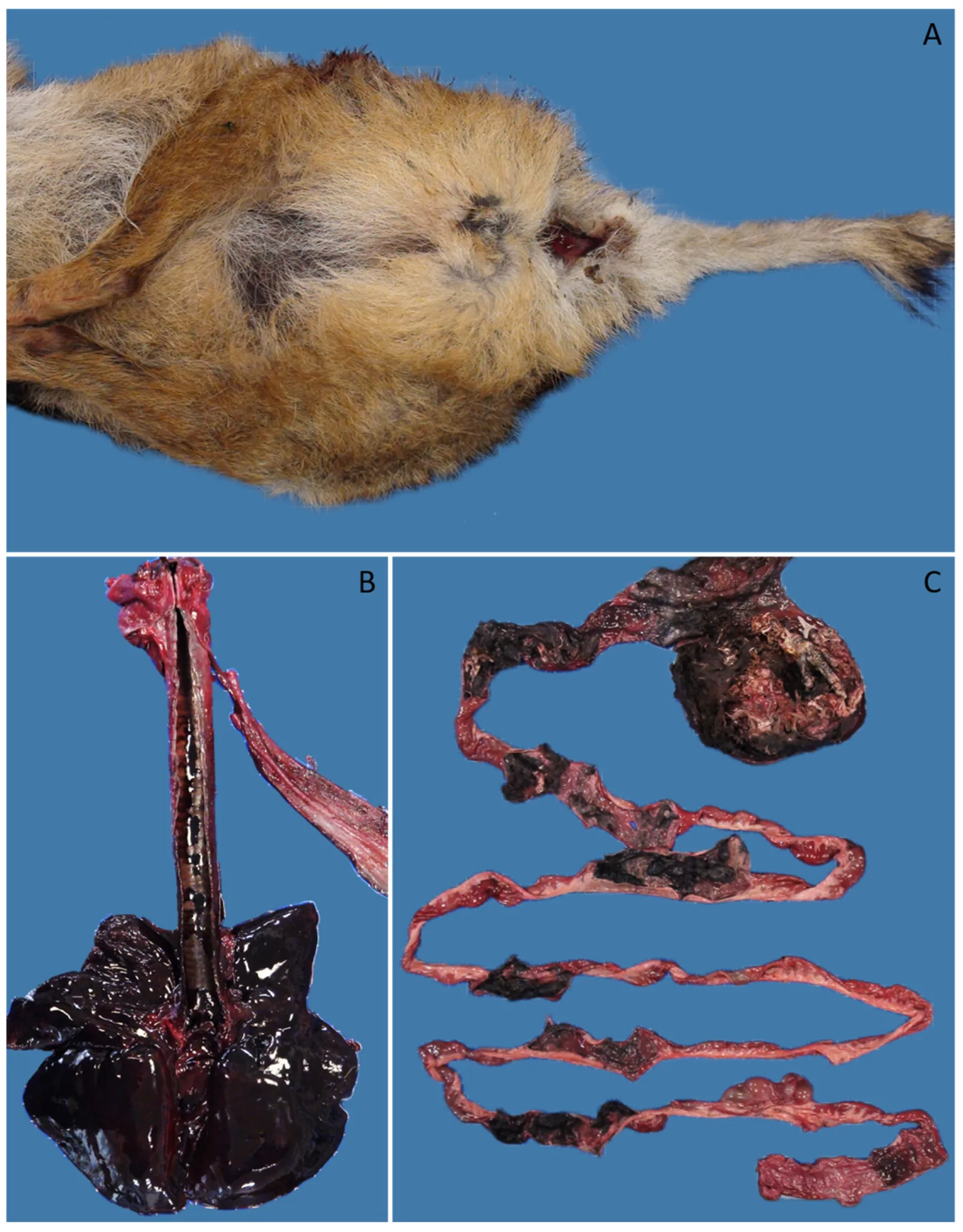
Necropsy findings in the golden jackal (Subject 2). (**A**) Perineal region in dorsal decubitus; the fur is soiled with mucoid, dark pink-to-brick-red fecal material. (**B**) Esophagus, trachea, and lungs. The esophageal lumen contains scant, mucoid, brick-red material. The tracheal lumen contains a moderate amount of blood-tinged fluid, with initial gray-green mucosal discoloration secondary to autolysis. Lungs are edematous and diffusely congested (dark red-to-black parenchyma) with signs of advanced autolysis. (**C**) Macroscopic view of the gastrointestinal tract. The stomach is distended by abundant content, including identifiable remains of two rodents and avian bones. Small intestinal content is creamy and dark red-to-black, transitioning into unformed, mucoid, dark pink-to-red material in the large intestine.

A sub-adult female golden jackal (designated as “Subject 2”) from the Autonomous Province of Bolzano was found on the side of a provincial road. On presentation, the animal was in good nutritional condition and showed pronounced post-mortem changes. External examination revealed mild tick infestation, slight congestion of the visible mucous membranes, and moderate perineal staining with dark pink to red, mucoid feces (Figure 4A). On subcutaneous examination, locally extensive hemorrhagic suffusions were present, deeply involving the muscular planes of the left thoracic region and of the ventrolateral abdomen along the linea alba. The thoracic cavity contained an abundant, uncoagulated serohemorrhagic fluid (hemothorax).

In the respiratory and cardiovascular systems, the tracheal lumen contained a moderate amount of bloody fluid. The lungs were edematous and diffusely congested, with multifocal to coalescing dark red-to-black discoloration of the parenchyma (Figure 4B). The left atrioventricular valve showed mild edematous thickening. Moderate macroscopic changes were present throughout the gastrointestinal tract (Figure 4C). The stomach was distended by abundant content, including the identifiable remains of two rodents and avian bones. The small intestine contained creamy, dark red-to-black material, which became unformed, mucoid, and dark pink to red in the large intestine. Throughout the gastrointestinal tract, the mucosa could not be adequately evaluated because of advanced post-mortem changes. The liver was moderately congested and markedly autolytic. In the lymphopoietic and urogenital systems, gross alterations included congestion of the thymus and lymph nodes, with the regional lymph nodes appearing moderately reactive, whereas the Peyer’s patches could not be reliably evaluated because of autolysis. The kidneys were heavily affected by post-mortem autolysis and their capsule stripped easily, although diffuse parenchymal congestion was still evident at the corticomedullary junction. The urinary bladder was empty.

Molecular analyses detected RVA in samples from the ileum, mesenteric lymph node, lung, and oropharyngeal swab.

## 4. Discussion

The aim of this study was to determine whether wild-canid cadavers collected through passive wildlife surveillance, under official oversight and supported by forensic pathology examination, could represent a robust and informative source for assessing the circulation of emerging viruses in expanding carnivore populations.

Among the gross pathology findings, the absence of significant associations between viral positivity and pathological findings should be interpreted with caution because several factors markedly compromised the quality and interpretability of the pathological findings: convenience sampling inherent to passive surveillance, frequent polytrauma from vehicle collisions, prolonged post-mortem intervals before carcass recovery, and routine freezing at −20 °C prior to necropsy. In addition, in accordance with institutional biosecurity protocols, carcasses of wild canids included in passive wildlife-surveillance programs undergo rabies-exclusion testing at the National Reference Centre for Rabies (IZSVe) prior to necropsy. This additional step resulted in further delays in the examination of animals, contributing to autolytic changes and freezing artifacts that compromised the proper pathological assessment of several subjects.

Gross lesions suggestive of infectious processes were often obscured by severe trauma (polytrauma, gunshot wounds) or by systemic hemorrhage in poisoned animals [56]. Furthermore, the gastrointestinal tract, primary site of replication for the viruses investigated, undergoes rapid autolysis, further limiting the evaluation of enteric changes [57]. The spatial distribution of wild canid carcasses was also biased: severely debilitated animals are less likely to leave their habitat and may be under-represented, whereas individuals with subclinical or mild infections interact more often with anthropogenic features, increasing their risk of vehicle collisions and subsequent submission [58,59]. These factors may explain viral detection without evident lesions. Macroscopic examination of the digestive and hemolymphopoietic systems showed no significant association between viral positivity and pathological findings, since the alterations were non-specific and confounded by age, carcass preservation, unassessed concurrent infections, and lesions related to the cause of death.

### 4.1. Cases Description

Regarding Case 1, the severity of the gastroenteritis and lymphopoietic lesions, together with the detection of concurrent viral infections and the exclusion of traumatic causes, supported the diagnosis of distributive shock secondary to acute hemorrhagic gastroenteritis [60,61]. The concurrent infection with RVA, CPV-2b and CCoV suggests that multiple viral infections may have contributed to the clinical and pathological presentation [62,63].

For Case 2, the presence of severe hemothorax and pulmonary hemorrhages associated with polytrauma indicated hypovolemic shock with acute respiratory failure as the primary cause of death. Nonetheless, molecular analyses demonstrated systemic dissemination of RVA at the time of death, despite its predominantly enteric tropism [25,64,65]. This systemic involvement may have contributed both to the animal’s use of roads as energetically favorable routes and to a reduced ability to perceive the risk posed by oncoming vehicles, thereby increasing the likelihood of collision [66,67].

### 4.2. Emerging Viruses in Gray Wolves and Golden Jackals

MRV was detected in 27.71% of gray wolves and 33.33% of golden jackals, representing the first report of the virus in both species. In both hosts, the distribution of positive matrices suggests a viral cycle primarily involving the gastrointestinal tract [38,48,68], with subsequent involvement of associated lymphoid tissues. The detection of MRV in mesenteric lymph nodes, occasionally as the only positive matrix, suggests active viral involvement rather than merely passive transit through the gastrointestinal tract following ingestion of infected prey species, such as wild boar or wild ruminants, in which MRV circulation has previously been documented [69]. The recurrent detection of MRV in ileal tissue, despite only two positive rectal swabs, suggests active yet potentially intermittent fecal shedding and indirect environmental transmission. The successful isolation of MRV from both a gray wolf and a golden jackal represents a key advancement toward the characterization of circulating strains. Ongoing genomic analyses will enable detailed strain characterization and assessment of genetic relationships with MRV variants detected in sympatric and non-sympatric hosts. These data will contribute to clarifying potential reassortment events and the likelihood of interspecies transmission involving domestic animals and humans.

The prevalence of RVA was markedly higher than that of MRV in both gray wolves (60.24%) and golden jackals (61.11%). To date, no data are available on RVA circulation in gray wolves. In golden jackals, the only previous molecular study, conducted on fecal samples from a larger population, reported a lower prevalence of 36.6% [25]. RVA was consistently detected in the ileum and mesenteric lymph nodes, whereas all rectal swabs tested negative. The failure to detect viruses in rectal swabs, despite frequent positivity in intestinal tissues, may result from intermittent shedding or from differences in sample preservation and assay sensitivity [45,70]. Virus isolation attempts were unsuccessful, which is not unexpected considering the fastidious growth requirements of RVA in cell cultures and the difficulty of isolating rotaviruses directly from clinical specimens [71]. Moreover, the infectivity of field-collected samples may be affected by pre-analytical factors, including the time elapsed between collection, processing and storage conditions, which may further reduce the likelihood of successful virus recovery. Future virus isolation and genomic characterization will be essential to investigate their genetic diversity, assess potential reassortment with strains infecting domestic and wild hosts, and improve our understanding of their epidemiological and pathogenic significance at the wildlife–domestic interface.

### 4.3. Circulation of Established Viral Pathogens in Gray Wolves and Golden Jackals

Molecular analyses for both enteric and respiratory CCoV forms were performed on lung and ileum samples, and no evidence of infection was found in the golden jackals examined. This may suggest that viral circulation in this species is absent or limited in the study area, but the probability of freedom from infection cannot be reliably estimated. The sample size was too small to provide adequate statistical power, and published molecular data in golden jackals are scarce. The only available study (Kureljušić et al., 2026) found no positive animals among 60 jackals in Serbia, but it was based on pooled lung and lymph node samples [72]. Finally, the unknown size of the local golden jackal population precludes the use of finite-population approaches to estimate the probability of freedom from infection. These results therefore indicate only the absence of detected infection in the sampled animals, not the absence of CCoV in the local population, and larger, more representative samples are needed to clarify the role of golden jackals in CCoV circulation.

In gray wolves, no evidence of canine respiratory coronavirus was detected. Conversely, enteric CCoV was detected in 12.5% of gray wolves, representing a higher prevalence than that previously reported by Reggiani et al. (2026) with a similar opportunistic sampling and by other studies in fecal samples collected from the Apennine wolf population [27,32]. Differences in sampling strategies, biological matrices, and molecular assays should be considered when comparing detection rates across studies. Overall, the pathogenic role of CCoV in free-ranging carnivores remains incompletely understood: most detections occur in asymptomatic animals, whereas in domestic dogs the virus has been documented in co-infections and associated with enteric or vascular syndromes [26,72]. CCoV could contribute to disease in hosts under stress or with concurrent infections, although this has not yet been demonstrated in free-ranging carnivores. Environmental transmission, through fecal contamination of shared habitats or scavenging of infected carcasses, is plausible in alpine ecosystems and may facilitate viral spread among sympatric carnivores [46].

In this study, CPV-2 was detected in 10.8% of gray wolves and 35.3% of golden jackals. In gray wolves, reported prevalence varies considerably across Europe depending on the study area; however, the prevalence observed in this study was lower than that reported in wild carnivores in other European [73,74] and Italian investigations within the Apennines mountain range [75,76]. This difference may be partly methodological, as only ileum samples were analyzed here, whereas previous studies also examined tissues such as spleen, lymph nodes, and tongue. It may also be ecological: most Italian studies focused on Apennine wolf populations, which live in areas with a higher density of free-ranging domestic dogs than north-eastern Italy, possibly favoring viral transmission and higher infection rates. In golden jackals, CPV-2 prevalence has previously been reported at 4.4% in the Serbian populations [30], whereas Leopardi et al. (2022) reported a 25% molecular detection rate for *Protoparvovirus carnivoran* 1 in golden jackals from north-eastern Italy, with all positive samples identified as FPV [29], although the latter study was based on a small sample size. In this study, CPV-2 positivity appeared numerically higher in sub-adults (50.0%) than in adults (16.7%) and juveniles (33.3%); however, the limited sample size and the wide confidence intervals preclude any reliable interpretation of this trend. Nevertheless, the potential pathological relevance of this virus in free-ranging carnivores should not be overlooked. In the absence of vaccination, variation in natural exposure may influence population-level immunity and the proportion of susceptible individuals, potentially affecting the risk of outbreaks and disease-associated mortality [77].

Variant characterization revealed the concurrent circulation of CPV-2a, CPV-2b, CPV-2c, and original CPV-2 in gray wolves. The detection of multiple CPV-2 variants may reflect repeated introductions and/or circulation between domestic and wild carnivore populations. This pattern is consistent with previous evidence indicating frequent virus exchange at the wildlife–domestic interface and the capacity of CPV-2 variants to circulate across multiple domestic and wild carnivore species [28,78]. Previous Italian studies showed that genetically related CPV-2 strains circulate in both domestic dogs and gray wolves, supporting a shared viral pool rather than a separate wildlife-specific population [78]. More recent investigations in Italian wild carnivores have likewise provided evidence of multiple CPV-2 lineages and patterns compatible with cross-species spillover at the domestic–wildlife interface [28].

Interestingly, all golden jackal samples initially classified as original CPV-2 by FMCA and successfully sequenced were subsequently identified as FPV. This finding highlights the limitations of variant-specific screening assays when dealing with closely related carnivore protoparvoviruses and emphasizes the importance of confirmatory sequencing. The detection of FPV in successfully sequenced golden jackals’ samples further supports the ability of this virus to infect a broad range of non-felid carnivore hosts, in line with previous reports in wild canids [79,80]. Although the epidemiological significance of FPV infection in golden jackals remains unclear, these findings suggest that this species may participate in the ecology of FPV and highlight the potential for viral exchange at the wildlife–domestic animal interface, particularly where susceptible wild and domestic carnivore populations overlap [28,30,79].

This study has several limitations. The animals were not selected through an ad hoc sampling design but came from ongoing passive surveillance programs, so the demographic composition of the sample reflects the circumstances in which animals were detected and submitted. Together with the limited sample size and the marked imbalance between gray wolves (*n* = 83) and golden jackals (*n* = 18), this produced subgroups with low expected cell counts and may have reduced the statistical power to detect associations. Passive surveillance also likely skewed age and sex distribution, with sub-adults, particularly males, over-represented because dispersal behavior exposes them to road mortality [81,82,83,84]. Despite these limitations, investigating multiple viruses at once provides essential baseline data on pathogens that are poorly and not recently studied in wild canids. Although transmission pathways cannot be inferred, the detection of potentially emerging and zoonotic agents supports further research at the wildlife–domestic interface and sustained One Health surveillance.

## 5. Conclusions

This study documents the circulation of multiple viruses in expanding wild canid populations in north-eastern Italy and shows that passive surveillance supported by forensic gross pathology is a robust and informative tool for monitoring viral circulation in free-ranging carnivores. It provides the first evidence of MRV infection in gray wolves and golden jackals, including viral isolation, and a high detection rate of RVA, suggesting that this zoonotic virus is more widespread in wild canids than previously recognized. The detection of CCoV, different CPV-2 variants in wolves, and FPV in jackals highlights complex viral dynamics at the wildlife–domestic interface. Despite limitations related to passive sampling and carcass preservation, the findings underscore the value of wildlife health monitoring, and further surveillance and molecular characterization, especially of zoonotic agents such as MRV and RVA, are needed within a One Health framework.

## Figures and Tables

**Table 1:** Gross gastrointestinal and associated lymphoid tissue findings in gray wolves and golden jackals. Data are presented as the number of animals and the corresponding percentage for each category within each species. Abbreviations: *n*, total number of subjects examined.

	Species	
	Gray Wolf (*n* = 83)	Golden Jackal *(n* = 18)
Intestinal content consistency		
Formed	55—(66.3%)	14—(77.8%)
Poorly formed	21—(25.3%)	2—(11.1%)
Unformed	7—(8.4%)	2—(11.1%)
Lymphoid tissue findings		
Prominent Peyer’s patches	10—(8.3%)	2—(11.1%)
Mesenteric lymph nodes enlargement and/or congestion	16—(19.3%)	2—(11.1%)
Other gastrointestinal findings		
Intestinal cestodes	48—(57.8%)	2—(11.1%)

**Table 2:** Prevalence of mammalian orthoreovirus (MRV), rotavirus A (RVA), canine coronavirus (CCoV), canine parvovirus type 2 (CPV-2) in gray wolves (*Canis lupus*) and golden jackals (*Canis aureus*) stratified by species and age class. Values are expressed as number of positive animals/total tested with valid results and prevalence (%) with 95% confidence intervals (Wilson score method) in parentheses. Abbreviations: ADU, adult; SUB, sub-adult; JUV, juvenile.

Species	Age-Class	Pathogen			
		MRV	RVA	CCoV	CPV-2
Gray wolf	ADU	8/25—32.0% (17.2–51.6)	13/25—52.0% (33.5–70.0)	5/22—22.7% (10.1–43.4)	2/20—10.0% (2.8–30.1)
Gray wolf	JUV	2/14—14.3% (4.0–39.9)	8/14—57.1% (32.6–78.6)	1/14—7.1% (1.3–31.5)	1/14—7.1% (1.3–31.5)
Gray wolf	SUB	13/44—29.5% (18.2–44.2)	29/44—65.9% (51.1–78.1)	4/44—9.1% (3.6–21.2)	5/40—12.5% (5.5–26.1)
Golden jackal	ADU	3/6—50.0% (18.8–81.2)	4/6—66.7% (30.0–90.3)	0/6—0.0% (0.0–39.0)	1/6—16.7% (3.0–56.4)
Golden jackal	JUV	1/3—33.3% (6.1–79.2)	1/3—33.3% (6.1–79.2)	0/3—0.0% (0.0–56.1)	1/3—33.3% (6.1–79.2)
Golden jackal	SUB	2/9—22.2% (6.3–54.7)	6/9—66.7% (35.4–87.9)	0/9—0.0% (0.0–29.9)	4/8—50.0% (21.5–78.5)

**Table 3:** Prevalence of mammalian orthoreovirus (MRV), rotavirus A (RVA), canine coronavirus (CCoV), and canine parvovirus type 2 (CPV-2) in gray wolves (*Canis lupus*) and golden jackals (*Canis aureus*) stratified by species and sex. Values are expressed as number of positive animals/total tested with valid results and prevalence (%) with 95% confidence intervals (Wilson score method) in parentheses. Abbreviations: F, female; M, male.

Species	Sex	Pathogen			
		MRV	RVA	CCoV	CPV-2
Gray wolf	F	9/30—30.0% (16.7–47.9)	20/30—66.7% (48.8–80.8)	3/30—10.0% (3.5–25.6)	3/27—11.1% (3.8–28.1)
Gray wolf	M	14/53—26.4% (16.4–39.6)	30/53—56.6% (43.3–69.0)	7/50—14.0% (6.9–26.2)	5/47—10.6% (4.6–22.6)
Golden jackal	F	2/8—25.0% (7.1–59.1)	6/8—75.0% (40.9–92.9)	0/8—0.0% (0.0–32.4)	2/7—28.6% (8.2–64.1)
Golden jackal	M	4/10—40.0% (16.8–68.7)	5/10—50.0% (23.7–76.3)	0/10—0.0% (0.0–27.8)	4/10—40.0% (16.8–68.7)

## Data Availability

The data supporting the findings of this study are available within the article. Requests for further details can be directed to the corresponding author.

## Supplementary Materials

The following supporting information can be downloaded at: https://www.mdpi.com/article/10.3390/ani16213499/s1, Table S1: Primers and probes used for molecular assays.

## References

[1] Daszak P., Cunningham A.A., Hyatt A.D. (2001). Anthropogenic Environmental Change and the Emergence of Infectious Diseases in Wildlife. Acta Trop..

[2] Marrana M., Prata J.C., Ribeiro A.I., Rocha-Santos T. (2022). Chapter 3—Epidemiology of Disease through the Interactions between Humans, Domestic Animals, and Wildlife. One Health.

[3] Carpio A.J., Apollonio M., Acevedo P. (2021). Wild Ungulate Overabundance in Europe: Contexts, Causes, Monitoring and Management Recommendations. Mammal Rev..

[4] Cimatti M., Ranc N., Benítez-López A., Maiorano L., Boitani L., Cagnacci F., Čengić M., Ciucci P., Huijbregts M.A.J., Krofel M. (2021). Large Carnivore Expansion in Europe Is Associated with Human Population Density and Land Cover Changes. Divers. Distrib..

[5] Mancinelli S., Falco M., Boitani L., Ciucci P. (2019). Social, Behavioural and Temporal Components of Wolf (*Canis lupus*) Responses to Anthropogenic Landscape Features in the Central Apennines, Italy. J. Zool..

[6] Boitani L., Linnell J.D.C., Pereira H.M., Navarro L.M. (2015). Bringing Large Mammals Back: Large Carnivores in Europe. Rewilding European Landscapes.

[7] Torretta E., Dondina O., Delfoco C., Riboldi L., Orioli V., Lapini L., Meriggi A. (2020). First Assessment of Habitat Suitability and Connectivity for the Golden Jackal in North-Eastern Italy. Mamm. Biol..

[8] Veronesi F., Deak G., Diakou A. (2023). Wild Mesocarnivores as Reservoirs of Endoparasites Causing Important Zoonoses and Emerging Bridging Infections across Europe. Pathogens.

[9] Taylor L.H., Latham S.M., Woolhouse M.E. (2001). Risk Factors for Human Disease Emergence. Philos. Trans. R. Soc. Lond. B Biol. Sci..

[10] Zimen E., Boitani L. (1975). Number and Distribution of Wolves in Italy. Z. Für Säugetierkd..

[11] La Morgia V., Marucco F., Aragno P., Salvatori V., Gervasi V., De Angelis D., Fabbri E., Caniglia R., Velli E., Avanzinelli E. (2022). Stima Della Distribuzione e Consistenza Del Lupo a Scala Nazionale 2020/2021.

[12] Marucco F., Boiani M.V., Dupont P., Milleret C., Avanzinelli E., Pilgrim K., Schwartz M.K., von Hardenberg A., Perrone D.S., Friard O.P. (2023). A Multidisciplinary Approach to Estimating Wolf Population Size for Long-Term Conservation. Conserv. Biol..

[13] Zanni M., Brogi R., Merli E., Apollonio M. (2023). The Wolf and the City: Insights on Wolves’ Conservation in the Anthropocene. Anim. Conserv..

[14] Cerri J., Musto C., Stefanini F.M., di Nicola U., Riganelli N., Fontana M.C., Rossi A., Garbarino C., Merialdi G., Ciuti F. (2023). A Human-Neutral Large Carnivore? No Patterns in the Body Mass of Gray Wolves across a Gradient of Anthropization. PLoS ONE.

[15] Life Wolfalps EU (2024). La distribuzione del lupo nelle regioni alpine 2020–2024.

[16] Bassi E., Pervan I., Ugarković D., Kavčić K., Maksan M.T., Krofel M., Šprem N. (2021). Attacks on Hunting Dogs: The Case of Wolf–Dog Interactions in Croatia. Eur. J. Wildl. Res..

[17] Franchini M., Corazzin M., Bovolenta S., Filacorda S. (2021). The Return of Large Carnivores and Extensive Farming Systems: A Review of Stakeholders’ Perception at an EU Level. Animals.

[18] Peltola T., Heikkilä J. (2015). Response—Ability in Wolf-Dog Conflicts. Eur. J. Wildl. Res..

[19] Suutarinen J., Kojola I. (2018). One Way or Another: Predictors of Wolf Poaching in a Legally Harvested Wolf Population. Anim. Conserv..

[20] Spassov N., Acosta-Pankov I. (2019). Dispersal History of the Golden Jackal (Canis Aureus Moreoticus Geoffroy, 1835) in Europe and Possible Causes of Its Recent Population Explosion. Biodivers. Data J..

[21] Trouwborst A., Krofel M., Linnell J.D.C. (2015). Legal Implications of Range Expansions in a Terrestrial Carnivore: The Case of the Golden Jackal (*Canis aureus*) in Europe. Biodivers. Conserv..

[22] Lapini L., Pecorella S., Villa M., Ferri M. (2021). Panoramica Aggiornata Delle Conoscenze Su Canis Aureus in Italia. Quad. Mus. Civ. Stor. Nat. Ferrara.

[23] Krofel M., Giannatos G., Ćirovič D., Stoyanov S., Newsome T. (2017). Golden Jackal Expansion in Europe: A Case of Mesopredator Release Triggered by Continent-Wide Wolf Persecution?. Hystrix Ital. J. Mammal..

[24] Ranc N., Wilmers C.C., Maiorano L., Warton D.I., Álvares F., Cagnacci F., Hatlauf J., Acosta-Pankov I., Banea O., Berce T. (2026). Human Shielding from Wolves Facilitates Jackal Expansion across Europe. Nat. Ecol. Evol..

[25] Kunić V., Barbić L., Šimić J., Mikuletič T., Kogoj R., Koritnik T., Steyer A., Konjević D., Bujanić M., Prišlin Šimac M. (2025). Interspecies Transmission and Genome Heterogeneity of Porcine-Originated Rotavirus A between Domestic Pigs and Wildlife in the Croatian Ecosystem. Sci. Total Environ..

[26] Alfano F., Fusco G., Mari V., Occhiogrosso L., Miletti G., Brunetti R., Galiero G., Desario C., Cirilli M., Decaro N. (2020). Circulation of Pantropic Canine Coronavirus in Autochthonous and Imported Dogs, Italy. Transbound. Emerg. Dis..

[27] Molnar B., Duchamp C., Möstl K., Diehl P.-A., Betschart B. (2014). Comparative Survey of Canine Parvovirus, Canine Distemper Virus and Canine Enteric Coronavirus Infection in Free-Ranging Wolves of Central Italy and South-Eastern France. Eur. J. Wildl. Res..

[28] Sarchese V., Di Profio F., Robetto S., Orusa R., Vuillermoz B., Pellegrini F., Marsilio F., Martella V., Di Martino B. (2025). Molecular Survey for Major Canine Enteric Viral Pathogens in Wild Carnivores, Northwestern Italy. Vet. Sci..

[29] Leopardi S., Milani A., Cocchi M., Bregoli M., Schivo A., Leardini S., Festa F., Pastori A., de Zan G., Gobbo F. (2022). Carnivore Protoparvovirus 1 (CPV-2 and FPV) Circulating in Wild Carnivores and in Puppies Illegally Imported into North-Eastern Italy. Viruses.

[30] Milićević V., Glišić D., Veljović L., Vasić A., Milovanović B., Kureljušić B., Paunović M. (2024). Protoparvovirus Carnivoran 1 Infection of Golden Jackals Canis Aureus in Serbia. Vet. Res. Commun..

[31] Moon B.-Y., Jang J., Kim S.-H., Kim Y.-H., Lee H.-K., So B., Park C.-K., Lee K.-K. (2020). Genetic Characterization of Canine Parvovirus Type 2c from Domestic Dogs in Korea. Transbound. Emerg. Dis..

[32] Ferraro E., Rold G.D., Celva R., Libera E.D., Leopardi S., Simonato G., Benedictis P.D., Cappai N., Dissegna A., Citterio C.V. (2026). Monitoring the Health of Wolves (*Canis lupus*): Integrating Conservation and Public Health. PLoS ONE.

[33] Giordano M.O., Martínez L.C., Isa M.B., Ferreyra L.J., Canna F., Paván J.V., Paez M., Notario R., Nates S.V. (2002). Twenty Year Study of the Occurrence of Reovirus Infection in Hospitalized Children with Acute Gastroenteritis in Argentina. Pediatr. Infect. Dis. J..

[34] Mikuletič T., Steyer A., Kotar T., Zorec T.M., Poljak M. (2019). A Novel Reassortant Mammalian Orthoreovirus with a Divergent S1 Genome Segment Identified in a Traveler with Diarrhea. Infect. Genet. Evol..

[35] Rosa U.A., Ribeiro G.D.O., Villanova F., Luchs A., Milagres F.A.D.P., Komninakis S.V., Tahmasebi R., Lobato M.C.A.B.S., Brustulin R., Chagas R.T.D. (2019). First Identification of Mammalian Orthoreovirus Type 3 by Gut Virome Analysis in Diarrheic Child in Brazil. Sci. Rep..

[36] Steyer A., Gutiérrez-Aguire I., Kolenc M., Koren S., Kutnjak D., Pokorn M., Poljšak-Prijatelj M., Rački N., Ravnikar M., Sagadin M. (2013). High Similarity of Novel Orthoreovirus Detected in a Child Hospitalized with Acute Gastroenteritis to Mammalian Orthoreoviruses Found in Bats in Europe. J. Clin. Microbiol..

[37] Lelli D., Beato M.S., Cavicchio L., Lavazza A., Chiapponi C., Leopardi S., Baioni L., De Benedictis P., Moreno A. (2016). First Identification of Mammalian Orthoreovirus Type 3 in Diarrheic Pigs in Europe. Virol. J..

[38] Decaro N., Campolo M., Desario C., Ricci D., Camero M., Lorusso E., Elia G., Lavazza A., Martella V., Buonavoglia C. (2005). Virological and Molecular Characterization of a Mammalian Orthoreovirus Type 3 Strain Isolated from a Dog in Italy. Vet. Microbiol..

[39] Chua K.B., Voon K., Crameri G., Tan H.S., Rosli J., McEachern J.A., Suluraju S., Yu M., Wang L.-F. (2008). Identification and Characterization of a New Orthoreovirus from Patients with Acute Respiratory Infections. PLoS ONE.

[40] Chua K.B., Voon K., Yu M., Keniscope C., Abdul Rasid K., Wang L.-F. (2011). Investigation of a Potential Zoonotic Transmission of Orthoreovirus Associated with Acute Influenza-Like Illness in an Adult Patient. PLoS ONE.

[41] Kurogi H., Inaba Y., Tanaka Y., Ito Y., Sato K., Omori T. (1976). Isolation and Properties of Reovirus from Cattle in an Outbreak of Acute Respiratory Disease. Natl. Inst. Anim. Health Q.

[42] Ouattara L. (2011). Novel Human Reovirus Isolated from Children with Acute Necrotizing Encephalopathy. Emerg. Infect. Dis..

[43] Tyler K.L., Barton E.S., Ibach M.L., Robinson C., Campbell J.A., O’Donnell S.M., Valyi-Nagy T., Clarke P., Wetzel J.D., Dermody T.S. (2004). Isolation and Molecular Characterization of a Novel Type 3 Reovirus from a Child with Meningitis. J. Infect. Dis..

[44] Hermann L., Embree J., Hazelton P., Wells B., Coombs R.T.K. (2004). Reovirus Type 2 Isolated from Cerebrospinal Fluid. Pediatr. Infect. Dis. J..

[45] Estes M.K., Greenberg H.B. (2013). Rotaviruses. Fields Virology.

[46] Decaro N., Buonavoglia C. (2008). An Update on Canine Coronaviruses: Viral Evolution and Pathobiology. Vet. Microbiol..

[47] Tuteja D., Banu K., Mondal B. (2022). Canine Parvovirology—A Brief Updated Review on Structural Biology, Occurrence, Pathogenesis, Clinical Diagnosis, Treatment and Prevention. Comp. Immunol. Microbiol. Infect. Dis..

[48] Campalto M., Mazzotta E., Crescenti F., Carrino M., Fincato A., Danesi P., Cavicchio L., Gagliazzo L., Ceglie L., Natale A. (2026). Molecular Survey of Mammalian Orthoreovirus and Rotavirus A in Italian Dogs and Cats. Front. Vet. Sci..

[49] Brasington T., Hadley J., Stahler D., Stahler E., Cassidy K. (2023). A Visual Guide to Wolf Dentition and Age Determination: For Researchers and Wildlife Professionals. Wildl. Biol..

[50] Paciello O., Fico R. (2018). Linee Guida Nazionali per Le Autopsie a Scopo Forense in Medicina Veterinaria.

[51] Hoffmann B., Depner K., Schirrmeier H., Beer M. (2006). A Universal Heterologous Internal Control System for Duplex Real-Time RT-PCR Assays Used in a Detection System for Pestiviruses. J. Virol. Methods.

[52] Carrino M., Tassoni L., Campalto M., Cavicchio L., Mion M., Corrò M., Natale A., Beato M.S. (2022). Molecular Investigation of Recent Canine Parvovirus-2 (CPV-2) in Italy Revealed Distinct Clustering. Viruses.

[53] Liu Z., Bingga G., Zhang C., Shao J., Shen H., Sun J., Zhang J. (2019). Application of Duplex Fluorescence Melting Curve Analysis (FMCA) to Identify Canine Parvovirus Type 2 Variants. Front. Microbiol..

[54] Tucciarone C.M., Franzo G., Mazzetto E., Legnardi M., Caldin M., Furlanello T., Cecchinato M., Drigo M. (2018). Molecular Insight into Italian Canine Parvovirus Heterogeneity and Comparison with the Worldwide Scenario. Infect. Genet. Evol..

[55] Kumar S., Stecher G., Suleski M., Sanderford M., Sharma S., Tamura K. (2024). MEGA12: Molecular Evolutionary Genetic Analysis Version 12 for Adaptive and Green Computing. Mol. Biol. Evol..

[56] Gwaltney-Brant S.M. (2016). Veterinary Forensic Toxicology. Vet. Pathol..

[57] Novakoski E., Branchini G., Franco de Oliveira T., Flôres Kuhn J., Bueno Motter S., Lee P., Krauspenhar Rossato C., Frau Alveal V.A., Bordignon Nunes F. (2023). Time since Death: The Histological Chronotanatognostic. J. Forensic. Leg. Med..

[58] Lachish S., Murray K.A. (2018). The Certainty of Uncertainty: Potential Sources of Bias and Imprecision in Disease Ecology Studies. Front. Vet. Sci..

[59] Millán J., López-Bao J.V., García E.J., Oleaga Á., Llaneza L., Palacios V., de la Torre A., Rodríguez A., Dubovi E.J., Esperón F. (2016). Patterns of Exposure of Iberian Wolves (*Canis lupus*) to Canine Viruses in Human-Dominated Landscapes. Ecohealth.

[60] Mazzaferro E.M. (2020). Update on Canine Parvoviral Enteritis. Vet. Clin. N. Am. Small Anim. Pract..

[61] Steinel A., Parrish C.R., Bloom M.E., Truyen U. (2001). Parvovirus Infections in Wild Carnivores. J. Wildl. Dis..

[62] Pratelli A., Tempesta M., Roperto F.P., Sagazio P., Carmichael L., Buonavoglia C. (1999). Fatal Coronavirus Infection in Puppies Following Canine Parvovirus 2b Infection. J. Vet. Diagn. Investig..

[63] Castro T.X., Cubel Garcia R.d.C.N., Gonçalves L.P.S., Costa E.M., Marcello G.C.G., Labarthe N.V., Mendes-de-Almeida F. (2013). Clinical, Hematological, and Biochemical Findings in Puppies with Coronavirus and Parvovirus Enteritis. Can. Vet. J..

[64] Caddy S., Papa G., Borodavka A., Desselberger U. (2021). Rotavirus Research: 2014–2020. Virus. Res..

[65] Azevedo L.S., Costa F.F., Ghani M.B.A., Viana E., França Y., Medeiros R.S., Guiducci R., Morillo S.G., Primo D., Lopes R.D. (2023). Full Genotype Characterization of Brazilian Canine G3P [3] Strains during a 10-Year Survey (2012–2021) of Rotavirus Infection in Domestic Dogs and Cats. Arch. Virol..

[66] Calabuig C., Dantas A., Katzenberger M., Souza H., Sombra C., Megid J., Antunes J.M.A.d.P. (2020). Assessment of Rabies and Canine Distemper Viruses in Road-Killed Wildlife Mammals From the Semiarid Region of Northeastern Brazil. Trop. Conserv. Sci..

[67] Namroodi S., Gholami A., Shariat-Bahadori E. (2016). Toxoplasmosis May Lead to Road Kills of Persian Leopards (Panthera Pardus Saxicolor) in Golestan National Park, Iran. J. Wildl. Dis..

[68] Mazzotta E., Lucchese L., Corrò M., Ceglie L., Danesi P., Capello K., Natale A. (2024). Zoonoses in Dog and Cat Shelters in North-East Italy: Update on Emerging, Neglected and Known Zoonotic Agents. Front. Vet. Sci..

[69] Arnaboldi S., Righi F., Filipello V., Trogu T., Lelli D., Bianchi A., Bonardi S., Pavoni E., Bertasi B., Lavazza A. (2021). Mammalian Orthoreovirus (MRV) Is Widespread in Wild Ungulates of Northern Italy. Viruses.

[70] Martella V., Bányai K., Matthijnssens J., Buonavoglia C., Ciarlet M. (2010). Zoonotic Aspects of Rotaviruses. Vet. Microbiol..

[71] Arnold M., Patton J.T., McDonald S.M. (2009). Culturing, Storage, and Quantification of Rotaviruses. CP Microbiol..

[72] Kureljušić B., Milićević V., Glišić D., Vasić A., Savić B., Jezdimirović N., Erünlü N.G.O., Blasi B., Weissenbacher-Lang C. (2026). Retrospective Analysis of Possible Associations between Pneumocystis Spp. and Five Immunosuppressive Viral Pathogens in Three Wild Carnivore Species. BMC Vet. Res..

[73] Decaro N., Buonavoglia C. (2011). Canine Coronavirus: Not Only an Enteric Pathogen. Vet. Clin. N. Am. Small Anim. Pract..

[74] Calatayud O., Esperón F., Velarde R., Oleaga Á., Llaneza L., Ribas A., Negre N., de la Torre A., Rodríguez A., Millán J. (2020). Genetic Characterization of Carnivore Parvoviruses in Spanish Wildlife Reveals Domestic Dog and Cat-Related Sequences. Transbound. Emerg. Dis..

[75] Rosa G.M., Santos N., Grøndahl-Rosado R., Fonseca F.P., Tavares L., Neto I., Cartaxeiro C., Duarte A. (2020). Unveiling Patterns of Viral Pathogen Infection in Free-Ranging Carnivores of Northern Portugal Using a Complementary Methodological Approach. Comp. Immunol. Microbiol. Infect Dis..

[76] Balboni A., Urbani L., Delogu M., Musto C., Fontana M.C., Merialdi G., Lucifora G., Terrusi A., Dondi F., Battilani M. (2021). Integrated Use of Molecular Techniques to Detect and Genetically Characterise DNA Viruses in Italian Wolves (*Canis lupus* Italicus). Animals.

[77] Ndiana L.A., Lanave G., Desario C., Berjaoui S., Alfano F., Puglia I., Fusco G., Colaianni M.L., Vincifori G., Camarda A. (2021). Circulation of Diverse Protoparvoviruses in Wild Carnivores, Italy. Transbound. Emerg. Dis..

[78] Mech L.D., Goyal S.M., Paul W.J., Newton W.E. (2008). Demographic Effects of Canine Parvovirus on a Free-Ranging Wolf Population over 30 Years. J. Wildl. Dis..

[79] Battilani M., Scagliarini A., Tisato E., Turilli C., Jacoboni I., Casadio R., Prosperi S. (2001). Analysis of Canine Parvovirus Sequences from Wolves and Dogs Isolated in Italy. J. Gen. Virol..

[80] Coric I., Miletic G., Konjevic D., Boskovic I., Bujanic M., Skrinjaric A., Kovac S., Barbic L., Jungic A., Stevanovic V. (2026). Detection and Molecular Characterisation of Protoparvovirus Carnivoran1 in Golden Jackals (*Canis aureus*) in Croatia. Viruses.

[81] Calatayud O., Esperón F., Cleaveland S., Biek R., Keyyu J., Eblate E., Neves E., Lembo T., Lankester F. (2019). Carnivore Parvovirus Ecology in the Serengeti Ecosystem: Vaccine Strains Circulating and New Host Species Identified. J. Virol..

[82] Morales-González A., Fernández-Gil A., Quevedo M., Revilla E. (2022). Patterns and Determinants of Dispersal in Grey Wolves (*Canis lupus*). Biol. Rev. Camb. Philos. Soc..

[83] Mech L.D., Boitani L. (2019). Wolves: Behavior, Ecology, and Conservation.

[84] Lanszki J., Schally G., Heltai M., Ranc N. (2018). Golden Jackal Expansion in Europe: First Telemetry Evidence of a Natal Dispersal. Mamm. Biol..

