# Supplementary material for: Wildlife Surveillance in North-Eastern Italy: Diagnostic Insights and Viral Detection in Gray Wolves and Golden Jackals
Source: Animals (Basel). 2026 Nov 5;16(21):3499. doi: 10.3390/ani16213499 (PMC13762247; doi:10.3390/ani16213499)
Supplement: Supplementary file 1 [file animals-16-03499-s001.zip › animals-4631052-supplementary.pdf]

Table S1. Primers and probes used for molecular assays.

Pathogen	Primers/Probe identification	Primers/Probe sequence	Target	Amplicon size (bp)	Reference
MRV	MRV L1 Probe	5'- 6-FAM - TGGCAGCGDTGGATHCGBTATTC - BHQ-1 -3'	L1	136	Modified Wang et al., 2021 - https://doi.org/10.1080/22221751.2021.1933608
	MRV L1 For	5′-GCGAAYTCTTCAGCRGARGAGC-3′			
	MRV L1 Rev	5′-CGTGARAAAGCRCAGCATRRAGCC-3′			
RVA	RVA7 Probe	5'- Cy5 - ATAGTTAAAAGCTAACACTGTCAAAAACCTAAA - BBQ-650 -3'	NSP3	77	Otto et al., 2015 - http://dx.doi.org/10.1016/j.vetmic.2015.07.021<br>Pang et al., 2004 - https://doi.org/10.1002/jmv.20009
	RVA7-1F	5′-RCATRACCCYCTATGAGCAC-3′			
	RVA NVP3-R	5′- GGTCACATAACGCCCC-3′			
Exogenous internal positive control (Intype RNA)	EGFP-HEX	5′-HEX-AGCACCCAGTCCGCCCTGAGCA-BHQ1-3′	-	132	Hoffmann et al., 2006 - https://doi.org/10.1016/j.jviromet.2006.05.020
	EGFP-1-F	5′-GACCACTACCAGCAGAACAC-3′			
	EGFP-2-R	5′-GAACTCCAGCAGGACCATG-3′			
CPV-2/FPV	CPV-2 Probe	5′-FAM-TGGTCCTTTAACTGCATTAAATAATGTACC- TAMRA-3′	VP2	93	Carrino et al., 2022 - https://doi.org/10.3390/v14050917
	CPV-2 For	5′-GATCCAATTGGAGGTAAAACAGG-3′			
	CPV-2 Rev	5′-TTCTTTATCCCAAATTTGACC-3′			
Exogenous internal positive control (Intype DNA)	EGFP-HEX	5′-HEX-AGCACCCAGTCCGCCCTGAGCA-BHQ1-3′	-	176	Hoffmann et al., 2006 - https://doi.org/10.1016/j.jviromet.2006.05.020
	EGFP-1-F	5′-GACCACTACCAGCAGAACAC-3′			
	EGFP-10-R	5′-CTTGTACAGCTCGTCCATGC-3′			
FMCA (CPV-2)	Probe P1	5′-HEX-TGTAAATAATATGGATAAAACTGC-BHQ-1-3′	VP2	110	Liu et al., 2019 - https://doi:10.3389/fmicb.2019.00419
	87f-1	5′-GAAATCACAGCAAACTCAAGC-3′			
	87r-jb	5′-GCATGARTATCATCTAAAGCC-3′			
	Probe P2	5′-6-FAM-CCTTCCTGTAACAAATGATAATGTATT-BHQ-1-3′		86	
	cpv-F2	5′-AGCACATCAAGATACAGGAAGATATCC-3′			
	ab-Rev	5′-CCAATTGGATCTGTTGGTAGCAATACA-3′			
